# Patients with hidradenitis suppurativa are associated with risk of new-onset glaucoma: a propensity-score-matched cohort study

**DOI:** 10.7150/ijms.95395

**Published:** 2024-08-13

**Authors:** Chen-Pi Li, Chen-Yu Lin, Hsin-Yo Lu, San-Ni Chen, Ru-Yin Tsai, Hui-Chin Chang, Shiu-Jau Chen, Shuo-Yan Gau

**Affiliations:** 1 Department of Nursing & Tungs' Taichung MetroHarbor Hospital, Taiwan.; 2 School of Medicine, Chung Shan Medical University, Taichung, Taiwan.; 3 Department of Ophthalmology, China Medical University Hospital, Taichung, Taiwan.; 4 School of Medicine, College of Medicine, China Medical University, Taichung, Taiwan.; 5 Department of Anatomy, School of Medicine, Chung Shan Medical University, Taichung, Taiwan; 6 Department of Medical Education, Chung Shan Medical University Hospital, Taichung, Taiwan.; 7 Evidence-based Medicine Center, Chung Shan Medical University Hospital, Taichung, Taiwan.; 8 Library, Chung Shan Medical University Hospital, Taichung, Taiwan.; 9 Department of Neurosurgery, Mackay Memorial Hospital, Taipei 10449, Taiwan.; 10 Department of Medicine, Mackay Medical College, New Taipei City 25245, Taiwan.; 11 Department and Graduate Institute of Business Administration, National Taiwan University, Taipei, Taiwan.; 12 Department of Pharmacology, Chung Shan Medical University, Taichung, Taiwan.; 13 Orthopedics Department, Chi-Mei Medical Center, Tainan, Taiwan.

**Keywords:** hidradenitis suppurativa, glaucoma, real-world study, electronic medical records

## Abstract

**Background:** Ocular comorbidities of hidradenitis suppurativa (HS) has been widely evaluated; however real-world evidence was scarce. Moreover, risk of glaucoma in HS patients remained unclear. This study aimed to evaluate the 5-year glaucoma risk in HS patients.

**Methods:** This retrospective cohort study used the TriNetX database covering 2005-2017. In total, 53,281 HS patients were propensity score matched 1:1 to controls based on demographics, including comorbidities, medications, healthcare utilization, etc. Patients were followed for 5 years post-index date. Glaucoma risks were calculated based on hazard ratios and 95% confidence intervals (95% CI). Stratified analyses by sex and age were performed.

**Results:** After matching, baseline characteristics were similar between groups. HS was associated with a 1.25 times higher 5-year glaucoma risk (95% CI, 1.10-1.42). The risk was significant within 1 year (HR=1.37; 95% CI, 1.03-1.82), 3 years (HR=1.31; 95% CI, 1.12-1.54), and 5 years post-index. In subgroup analysis, women had a 1.28 times higher risk (95% CI, 1.10-1.49). Patients aged 18-64 years (HR=1.33; 95% CI, 1.14-1.55) and ≥65 years (HR=1.33; 95% CI, 1.05-1.67) also presented elevated glaucoma risks.

**Conclusion:** This real-world data analysis demonstrated a significantly increased 5-year glaucoma risk in HS patients versus matched controls. Ocular complications should be concerned while managing HS patients.

## Introduction

Hidradenitis Suppurativa (HS), alternatively referred to as acne inversa and Verneuil's disease, represents a persistent inflammatory skin condition marked by recurring painful nodules, abscesses, and scarring. This condition predominantly affects areas rich in apocrine glands, including the axillae, breasts, groin, and perineum 1. The considerable psychological and emotional burden 2, coupled with physical comorbidities, can adversely affect patients' quality of life. The repercussions of this can result in a decrease in general health and a reduction in the quality of life, further compounding the already substantial effects of the disease 3. While the precise workings of HS remain partially understood, it is established that pro-inflammatory cytokines, such as tumor necrosis factor, IL-1β, and IL-17, are central to the progression of the illness 1.

Glaucoma is recognized as a complex and multifactorial progressive neurodegenerative disease characterized by a spectrum of clinical features, encompassing various risk factors and underlying mechanisms 4. Globally, its prevalence has been estimated at 3 to 4 percent among people aged 40-80 years, with projections indicating a significant increase in the burden of the disease, affecting around 111.8 million individuals by 2040, particularly affecting populations in Asia and Africa 5. This impending surge highlights the pressing need for a comprehensive understanding of the disease's origin and the formulation of effective treatments.

The prominent characteristics of glaucoma encompass degeneration of retinal ganglion cells (RGC), thinning of the retinal nerve fiber layer, and deformation of the optic disc 6. Its development primarily involves a complex interplay between increased intraocular pressure, vascular irregularities, neurodegeneration, and chronic inflammation 7. Neurodegeneration process can lead to a state described as para-inflammation, which denotes the adaptive reaction to detrimental stress or dysfunction. Intense para-inflammation can trigger the release of significant amounts of cytokines and chemokines, resulting in irreparable damage to the neural retina 4.

Although there is currently a lack of in-depth research exploring the relationship between HS and glaucoma, and glaucoma is seldom discussed as an ocular complication of HS 8, 9, both conditions are associated with mechanisms linked to inflammation. A recent study underscored the significant role of IL-38 in the immunopathology of chronic primary angle-closure glaucoma (CPACG) and HS 10, 11. Consequently, this investigation focused specifically on evaluating the 5-year risk of glaucoma in patients with HS. The expected outcome of this research is to improve the understanding of the interplay between these two diseases by the medical community, offering a valuable reference for future clinical diagnosis and treatment.

## Methods and Materials

This study was conducted retrospectively using de-identified data from the TriNetX research network, which is a global platform containing electronic medical records from over 120 healthcare organizations worldwide. The TriNetX collaborative network has been extensively used in previous studies to examine associations between exposures and outcomes 12-14. In this study, we accessed the United States-based network (US collaborative network) containing records from 60 healthcare organizations and approximately 87 million patients for analysis. To extract the baseline information of study participants, we identified respective ICD-10-CM for diseases identification and RxNorm for medication records (**Table S1**). This study was approved by the Institutional Review Board of Tungs' Taichung MetroHarbor Hospital (IRB TTMHH No.: 112208N).

The study population was selected from the TriNetX network by identifying patients aged 18 years or older with a documented history of HS between January 1, 2005 and December 31, 2017. Each patient was followed for a minimum of 5 years after the initial recorded HS diagnosis date, which was defined as the index date. Patients were excluded if they were deceased before the index date, had any glaucoma diagnosis or had any cancer diagnosis prior to the index date. A healthy control cohort was constructed by selecting patients who had undergone general medical examination within the study timeframe. Propensity score matching at a 1:1 ratio was implemented to match the HS and control cohorts based on demographic and clinical characteristics, including age, gender, substance use, race, comorbidities, comedications, lab results, socioeconomic factors, and prior healthcare utilization. After matching, the final analytic sample consisted of 53,281 HS patients and 53,281 matched controls.

We performed stratified analyses by age and sex to evaluate the HS-outcome association across various populations. Sensitivity analyses were also conducted using various matching algorithms (including: Crude model without matching; Model 1: propensity score matching performed on age at index and sex; Model 2: Propensity score matching was performed on age at index, sex, race and comorbidities; Model 3: Propensity score matching was performed on age at index, sex, race, comedications and substance use; Model 4: Propensity score matching was performed on age at index, sex, race, body mass index, status of comorbidities, comedication use, smoking, alcoholism and substance use, medical utilization status, lab data regarding inflammation status, socioeconomic status, and the use of corticosteroids) and washout periods (6 months, 12 months and 24 months). All analyses were executed using the TriNetX system. Hazard ratios and 95% confidence intervals were calculated where applicable. Propensity score matching utilized greedy nearest neighbor matching with a 0.1 caliper width. Standardized differences above 0.1 were considered statistically significant when assessing matched baseline characteristics between cohorts.

## Results

Some of the potential confounders, including age, sex, and socioeconomic issues, were significantly different between the HS group and controls. Before matching, people with HS were more likely to have comorbidities such as diabetes, migraine, and sleep apnea. Moreover, the ratio of substance uses and socioeconomic problems was also significantly higher in the HS group. After matching, differences between the two groups became insignificant (**Table 1**). In the current study, patients with HS were on average 33.6 years old and mostly white (44.7%).

**Figure 2:**
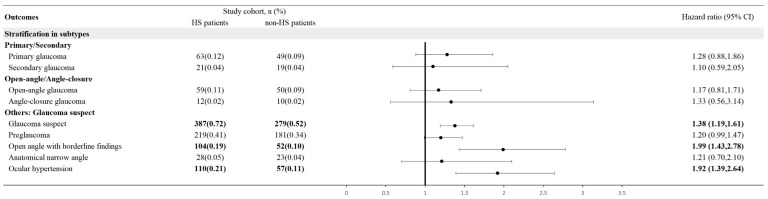
Forest plot: stratification in subtypes of glaucoma. Legends: 95% CI, 95% confidence interval; HS, hidradenitis suppurativa.

After matching on covariates, HS patients had a 37% higher risk of developing glaucoma in the first year after the index date compared to controls. The risk remained significantly elevated at 31% and 25% higher at 3 and 5 years after the index date (**Table 2**). Stratification analysis breaks down the risk by gender and age subgroups. Female HS patients had a statistically significant 28% higher risk of developing glaucoma within 5 years compared to female controls. However, the 5-year glaucoma risk for male HS patients was not significant compared to males without HS (HR, 1.21; 95% CI, 0.96, 1.52). Both young (18-64 years) and elderly (≥65 years) HS patients had around 30% higher risks of developing glaucoma within 5 years versus their age-matched controls (**Table 3**). In stratification based on subtypes, primary, secondary, open-angle and angle-closed glaucoma did not present statistically significant risk in HS patients. However, risk of glaucoma suspect (HR=1.38; 95% CI, 1.19-1.61), including ocular hypertension (HR=1.92; 95% CI, 1.39-2.64) and open angle with borderline findings (HR= 1.99; 95% CI, 1.43-2.78), were significantly increased in HS patients (**Figure 2**).

Using different propensity score matching variables, washout periods, HS patients consistently showed statistically significantly elevated hazards of developing glaucoma, with risks ranging from 19% to 90% higher than controls without HS (**Table S2**). While excluding all incident glaucoma occurring within 24 months after index date, the significance of HS-glaucoma association remained, with the HR of 1.23 (95% CI, 1.06,1.42) (**Table S3**).

## Discussion

Utilizing a global-federated, large-scale electronic medical records database, we observed that people with HS were associated with high risk of developing glaucoma, with the HR of 1.25 (95% CI, 1.10,1.42) in five-year follow up. The current study provided real-world evidence based on adjustment in critical covariates, and could serve as potential references for clinicians while managing HS patients with risk of ophthalmologic comorbidities. Several studies have previously suggested a strong relationship between HS and various inflammatory diseases 15-17. However, the possible association between HS and glaucoma is still unclear. Our study provides robust data, aiming to fill the evidence gap in this field.

HS is characterized by numerus comorbidities, including cardiovascular and renal disease, endocrine dysfunction, and a series of immune-mediated diseases 18. Pathogenesis of HS is associated with dysregulation of immune system. Hyperkeratosis of infundibular epithelium causes propagation of bacteria. This activates macrophages to secrete pro-inflammatory factors. IL-12 and IL-23 produced by macrophages stimulate TH1 and TH17 cells, which leads to strengthen the positive feedback of immune system. Due to bacterial propagation and immune dysregulation, follicle might be ruptured, which increases immune cell infiltration 19. This facilitates the elevation of IL-36γ from keratinocytes 20. A study indicated that IL-36γ induces G-CSF, a potential factor associated with HS lesions 21. Another study showed an elevation of IL-36α, IL-36β, IL-36γ and IL-36Ra in HS lesioned area, while IL-38 is increasing in both perilesional and lesioned area 22. Therefore, IL-36 and IL-38 play a crucial role in pathogenesis of HS.

Glaucoma is known to be a chronic eye disease, characterized by retinal ganglion cell atrophy with corresponding vision loss. Glaucoma can be categorized into various types, with primary open-angle glaucoma and primary angle-closure glaucoma being the two most prevalent forms. The pathogenesis of glaucoma is not well-established, involving intraocular pressure elevation, abnormal structural defect and blood flow, immune dysregulation, and low intracranial pressure 23. A recent study reveals potential correlation between IL-36, IL-37, IL-38, and chronic primary angle closure glaucoma (CPACG). Significant elevation of IL-36, IL-37 and IL-38 was detected in CPACG groups compared to age-related cataracts 24. IL-36 is commonly seen in various acute and chronic inflammatory disease, including skin, eye, kidney, liver, intestinal inflammation 25, 26. IL-38 is a potential anti-inflammatory cytokine, with extensive biological activity. It commonly involves in IL-1, IL-36 and TLR receptor pathway 27.

From pathogenesis of HS and glaucoma, it is evident that HS and glaucoma share a common mechanism involving IL-36 and IL-38 elevation. IL-36 is traditionally considered to be produced in various cells including keratinocytes, with fewer evidence related to eye structure 26. However, recent studies provide a novel insight into IL-36 as a potential factor to immune-mediated eye disease 24, 28. Therefore, dysregulation of IL-36-mediated immune pathway might be a possible factor for both HS and glaucoma.

Systemic inflammation in HS patients causes local upregulation of IL-36 and dysregulation of IL-38, which potentially leads to immune-mediated glaucoma. Under such circumstance, the use of steroid could serve as a potential confounding factor given that steroid use is related to glaucoma. However, as presented in the model 4 of Table S2, after adding steroid use as covariates for matching, the HS-glaucoma association remained. However, the definite mechanism of IL-36 and IL-38 in both HS and glaucoma is still unclear. Though IL-36 and IL-38 are elevated in both HS and CPACG, the observed association may be a result of chronic inflammation in both diseases. Further investigations are needed to provide conclusive evidence.

In addition, the HS-glaucoma association may potentially be affected by different sex groups. In our study, female HS patients had a higher risk than controls, while the risk of male patients was not significant compared to controls group. Females are considered one of the risk factors of glaucoma, and HS is also seemed to be female-predominant. Given the mechanisms and epidemiology of two diseases, we hypothesized that females may encounter a stronger inflammatory response, which elevates more IL-36 and IL-38 and in turn enhanced the risk of new-onset glaucoma. On the contrary, male patients may lack of enough responses, leading to absence of HS-glaucoma association.

Several limitations should be noted in our study. Firstly, due to the retrospective nature of this study, the causation of the observed association could not be established. Secondly, due to limitation of data, the definition of subtypes of glaucoma could be limited to misclassification bias as the definition was given based on administrative codes. Likewise, the data of HS patients' disease severity and treatment history were unavailable. As a result, we were incapable of assessing the influence of different HS stages on glaucoma. Third, in observational studies, issue of confounding bias should be prudently interpreted though propensity score matching has been performed 29, 30. Residual confounding bias may exist with possibility to affect our outcome. Consequently, the results of our study should be interpreted cautiously. Fourth, although stratified analyses and propensity score matching were performed on age at index, sex, race, and other factors of cohorts and controls in the current study, as in previous studies utilizing the US collaborative network in the TriNetX system 31, the generalizability to populations outside the United States may be limited and should be considered with caution. Given that previous studies have shown racial disparities in the epidemiology and pathophysiology of glaucoma, particularly the higher prevalence in Black and Hispanic populations 32, future studies should consider potential differences in the observed association between HS and glaucoma and focus on the effects of genetic variants and the diversity of living environments.

To sum up, our findings indicate an elevated risk of glaucoma in individuals with HS across various follow-up periods. This underscores the importance for clinicians to be mindful of ophthalmic comorbidities in HS patients when providing medical care. Glaucoma can negatively impact patients' quality of life 33. Early glaucoma screening could potentially reduce the comorbidity burden of HS patients, thereby preventing impairment in their quality of life. In this case, the clinical implication of our current study's findings is that we provide clinicians with real-world evidence regarding the early detection of ophthalmology-related comorbidities. Clinicians could integrate our findings into clinical practice, promote the collection of glaucoma-related family history, and result in the early detection of new-onset glaucoma. Current evidence could potentially serve as a basis for future comorbidity screening guidelines for HS.

## Supplementary Material

Supplementary tables.

## Figures and Tables

**Figure 1:**
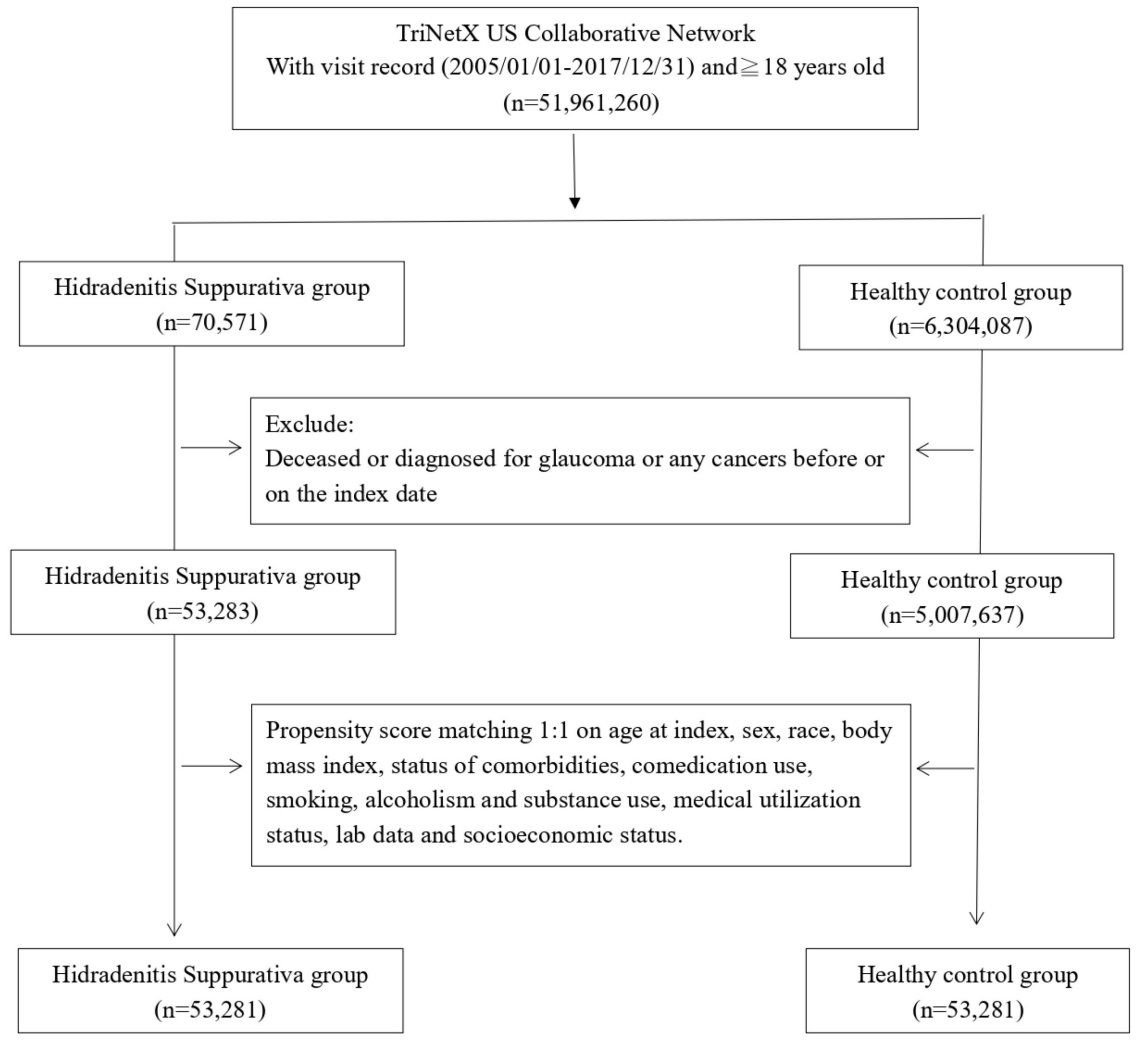
Patient selection process.

**Table 1:** Baseline characteristics of study subjects (before and after propensity score matching)

	Before matching				After matching^a^		
	HS cohort (n=53,283)	Control cohort (n= 5,007,637)	Std diff		HS cohort (n=53,281)	Control cohort (n=53,281)	Std diff
**Age at index**							
Mean ± SD	33.6±14.1	37.8±20.7	**0.23**		33.6±14.1	34.2±14.7	0.04
**Sex**							
Male	13347 (25.0)	2163477 (43.2)	**0.39**		13347 (25.1)	13376 (25.1)	0.00
Female	39555 (74.2)	2756615 (55.0)	**0.41**		39553 (74.2)	39501 (74.1)	0.00
**Race, n (%)**							
White	23835 (44.7)	3031232 (60.5)	**0.32**		23835 (44.7)	23832 (44.7)	0.00
Black or African American	18601 (34.9)	746722 (14.9)	**0.48**		18599 (34.9)	18854 (35.4)	0.01
Asian	852 (1.6)	172463 (3.4)	**0.12**		852 (1.6)	1523 (2.9)	0.09
American Indian or Alaska Native	239 (0.4)	16275 (0.3)	0.02		239 (0.4)	197 (0.4)	0.01
**Socioeconomic status**							
Socioeconomic/psychosocial circumstances problem	1020 (1.9)	35043 (0.7)	**0.11**		1019 (1.9)	920 (1.7)	0.01
**Lifestyle**							
Alcohol dependence, smoking and substance use	6320 (11.9)	161468 (3.2)	**0.33**		6318 (11.9)	6441 (12.1)	0.01
**Comorbidities**							
Hypertension	6673 (12.5)	488193 (9.7)	0.09		6673 (12.5)	6807 (12.8)	0.01
Diabetes mellitus	3897 (7.3)	202434 (4.0)	**0.14**		3896 (7.3)	3902 (7.3)	0.00
Hyperlipidemia	3524 (6.6)	321210 (6.4)	0.01		3524 (6.6)	3419 (6.4)	0.01
Migraine	2392 (4.5)	82403 (1.6)	**0.17**		2392 (4.5)	1979 (3.7)	0.04
Sleep apnea	2179 (4.1)	76427 (1.5)	**0.16**		2178 (4.1)	2151 (4.0)	0.00
**Medications**							
Promethazine	3939 (7.4)	145044 (2.9)	**0.20**		3937 (7.4)	3958 (7.4)	0.00
Ipratropium	1702 (3.2)	63227 (1.3)	**0.13**		1702 (3.2)	1635 (3.1)	0.01
Phenylephrine	1019 (1.9)	45038 (0.9)	0.09		1018 (1.9)	913 (1.7)	0.01
Ephedrine	733 (1.4)	28616 (0.6)	0.08		732 (1.4)	608 (1.1)	0.02
**Medical Utilization Status**							
Ambulatory visit	29843 (56.0)	2320931 (46.3)	**0.19**		29841 (56.0)	29989 (56.3)	0.01
Inpatient visit	8750 (16.4)	536300 (10.7)	**0.17**		8749 (16.4)	8723 (16.4)	0.00
**Laboratory data**							
BMI, n (%)							
≥ 35 (kg/m^2^)	4182 (7.8)	103063 (2.1)	**0.27**		4180 (7.8)	4315 (8.1)	0.01
C reactive protein, n (%)							
≥ 3 (mg/L)	2172 (4.1)	73078 (1.5)	**0.16**		2171 (4.1)	2162 (4.1)	0.00

HS: Hidradenitis Suppurativa; BMI, body mass index; SD, standardized difference ^a^ Propensity score matching was performed on age at index, sex, race, body mass index, status of comorbidities (including diabetes mellitus, hypertension, hyperlipidemia, migraines, sleep apnea), status of comedication use (phenylephrine, ephedrine, promethazine, ipratropium bromide), status of smoking, alcoholism and substance use, medical utilization status, lab data regarding inflammation status (CRP) and socioeconomic status.

**Table 2:** Risk of glaucoma under different follow-up time^a^

Outcomes	Hazard ratio (95% Confidence interval)^b^		
	1 year	3 years	5 years
Glaucoma	**1.37 (1.03, 1.82)**	**1.31 (1.12, 1.54)**	**1.25 (1.10, 1.42)**

HS: hidradenitis suppurativa ^a^Data present here were the value of follow up from 90 days after index date to the respective following up years. ^b^ Propensity score matching was performed on age at index, sex, race, body mass index, status of comorbidities (including diabetes mellitus, hypertension, hyperlipidemia, migraines, sleep apnea), status of comedication use (phenylephrine, ephedrine, promethazine, ipratropium bromide), status of smoking, alcoholism and substance use, medical utilization status, lab data regarding inflammation status (CRP) and socioeconomic status.

**Table 3:** Stratification analysis of glaucoma risk in HS patients

	Cases occurring new-onset glaucoma		
Subgroups	HS cohort (No. of event/ HS patient amount in each subgroup)	Control cohort (No. of event/ non-HS patient amount in each subgroup)	HR (95% CI)^a^
**Gender**			
Male	159/13345	132/13345	1.21 (0.96,1.52)
Female	383/39373	296/39373	**1.28 (1.10,1.49)**
**Age at index date**			
18-64 years old	382/48325	283/48325	**1.33 (1.14,1.55)**
≥ 65 years old	164/4953	126/4953	**1.33 (1.05,1.67)**

^a^ Propensity score matching was performed on age at index, sex, race, body mass index, status of comorbidities (including diabetes mellitus, hypertension, hyperlipidemia, migraines, sleep apnea), status of comedication use (phenylephrine, ephedrine, promethazine, ipratropium bromide), status of smoking, alcoholism and substance use, medical utilization status, lab data regarding inflammation status (CRP) and socioeconomic status.
