# Supplementary material for: Patients with hidradenitis suppurativa are associated with risk of new-onset glaucoma: a propensity-score-matched cohort study
Source: Int J Med Sci. 2024 Aug 13;21(11):2208–14. doi: 10.7150/ijms.95395 (PMC11373556; doi:10.7150/ijms.95395)
Supplement: Supplementary file 1 — Supplementary tables. [file ijmsv21p2208s1.pdf]

Supplementary Files

Supplementary Tables

Table S1. Utilized administrative codes in the current study

**Description**	**ICD-10-CM codes [a]**
Hidradenitis Suppurativa	L73.2
Neoplasms	C00-D49
Diabetes mellitus	E08-E13
Hypertension	I10
Hyperlipidemia	E78.5
Migraine	G43
Sleep apnea	G47.3
Socioeconomic and psychosocial circumstances	Z55-Z65
Substance abuse	F10-F19
Encounter for general examination	Z00
Glaucoma	H40
Primary glaucoma	H40.11, H40.2, Q15.0
Secondary glaucoma	H40.3-H40.6
Open-angle glaucoma	H40.1
Angle-closure glaucoma	H40.2
Glaucoma suspect	H40.0
Preglaucoma	H40.00
Open angle with borderline findings	H40.01-H40.02

Anatomical narrow angle	H40.03
Ocular hypertension	H40.05
**Medications**	**RxNorm/ ATC code**
Promethazine	RxNorm: 8745
Ipratropium	RxNorm: 7213
Phenylephrine	RxNorm: 8163
Ephedrine	RxNorm: 3966
Corticosteroids	ATC code: R01AD

[a]ICD-10-CM: International Classification of Diseases, Tenth Revision, Clinical Modification

Table S2. Sensitivity analysis: risk of glaucoma in HS patients based on different covariate matching models, with 5-year follow up

Outcomes	Hazard ratio (95% Confidence interval)				
	Crude	Model 1[a]	Model 2[b]	Model 3[c]	Model 4[d]
Glaucoma	**1.19 (1.10,1.30)**	**1.90 (1.64,2.20)**	**1.43 (1.26,1.64)**	**1.55 (1.35,1.77)**	**1.24 (1.09,1.41)**

[a] Propensity score matching was performed on age at index and sex

[b] Propensity score matching was performed on age at index, sex, race and comorbidities

[c] Propensity score matching was performed on age at index, sex, race, comedications and substance use

[d] Propensity score matching was performed on all matched covariates in the main analysis+ use of corticosteroids

Table S3. Sensitivity analysis: risk of glaucoma with different wash-out periods [a]

Outcomes	Hazard ratio (95% Confidence interval)[b]		
	6 months	12 months	24 months
Glaucoma	**1.25 (1.10,1.42)**	**1.23 (1.08,1.41)**	**1.23 (1.06,1.42)**

[a] Incident events occurred within each wash-out period were excluded in the corresponding analysis, following up for 5 years after index date

[b] Propensity score matching was performed on age at index, sex, race, body mass index, status of comorbidities (including diabetes mellitus, hypertension, hyperlipidemia, migraines, sleep apnea), status of comedication use (phenylephrine, ephedrine, promethazine, ipratropium bromide), status of smoking, alcoholism and substance use, medical utilization status, lab data regarding inflammation status (CRP) and socioeconomic status.
